# Biosynthesis of TiO_2_ nano particles by using Rosemary (*Rosmarinus officinalis*) leaf extracts and its application for crystal dye degradation under sunlight

**DOI:** 10.1186/s13065-024-01229-9

**Published:** 2024-06-29

**Authors:** Abel Saka, Leta Tesfaye Jule, Bayissa Badassa, Lamessa Gudata, N. Nagaprasad, R. Shanmugam, L. Priyanka Dwarampudi, Venkatesh Seenivasan, Krishnaraj Ramaswamy

**Affiliations:** 1 Department of Physics, College of Natural and Computational Science, Dambi Dollo University, Dambi Dollo, Ethiopia; 2 Centre for Excellence-Indigenous Knowledge, Innovative Technology Transfer and Entrepreneurship, Dambi Dollo University, Dambi Dollo, Ethiopia; 3 Ministry of innovation and technology, Dambi Dollo, Ethiopia; 4 Department of Mechanical Engineering, ULTRA College of Engineering and Technology, Madurai, 625 104 Tamil Nadu India; 5 Department of Pharmacognosy, TIFAC, CORE-HD, JSS College of Pharmacy, JSS Academy of Higher Education & Research, Ooty, Nilgiris, Tamil Nadu India; 6 Department of Pharmacognosy, JSS College of Pharmacy, JSS Academy of Higher Education & Research, Ooty, Nilgiris, Tamil Nadu India; 7 Department of Mechanical Engineering, Sri Eshwar College of Engineering, Coimbatore, India; 8 Department of Mechanical Engineering, Dambi Dollo University, Dambi Dollo, Ethiopia

**Keywords:** TiO_2_, NPs, Leaf extracts, Rosmarinus officinalis, Photo-degradation, Nanoparticles

## Abstract

Titanium dioxide (TiO_2_) nanoparticles were prepared through *Rosmarinus-officinalis* leaf extracts at 90 and 200°C. In this research, the degradations of methylene blues by using TiO_2_ nanoparticles Sun light radiations were studied. The synthesized materials were characterized using XRDs, UV-Vis, PL, SEM, TEM, EDS and XPS. The results displayed that bio-synthesis temperatures intrude the shapes and sizes of TiO_2_ nanoparticles. For TiO_2_-90, micrographs show separable crystalline with irregular morphologies and agglomerate cubic particles. For the other TiO_2_-200 sample, SEM and TEM micro-imaging shows crumbly agglomerated cubic structures. The XRD shows that the intense peaks observed at angles of 25.37°, 37.19°, 47.81° and 53.89° confirming a highly crystalline oriented as (004), (200), and (105) planes respectively. The optical properties of TiO2 nanoparticles synthesized were conveyed by PL and UV-Vis. The energy band gap calculated was 3.0 eV for both samples; that indicates heating temperature didn’t influence the band gap of the samples. The elemental composition Ti and O_2_ is shown by EDS and XPS. Photo-catalytic experiments discovered that TiO_2_-90 nanoparticles were well-organized in photo-degradations of MB, likened to TiO_2_-200. The great activities of TiO_2_-90 were because of better physicochemical characteristics associated with TiO_2_-200 effectively degrading MB under photo-light. Photo-degradations of dye under sunlight as plentifully obtainable energy sources by TiO_2_, synthesized by simpler techniques, can be hopeful to grow an eco-friendly and economical process.

## Introduction

Nanomaterial is manufactured its way into all features of survives; this material is actually progressively utilized in medicinal and therapeutic applications, cosmetic as well as personal products, energy storages and efficiencies, water treatments, air filtrations, ecological remediation, chemicals as well as biological sensor, military defense as well as explosive [1, 2] and in uncountable consumers yields and material. For occasion, in the areas of foods, nanomaterials can be utilized to deliver new palates and flavors; useful food; hygienic foods dispensation and wrapping; intelligence, light-weight, and strong packing; lengthy shelf lives; and concentrated agro-chemicals, colors, flavors, and preservative [3–5]. The explanations for using nanomaterials vary, rendering applications. Some solicitations can benefit from an increase in superficial areas per unit masses, which offers better functionalities [6]. Other utilizations can profit from the attainment of better controls of materials characteristics, developed dispersal and steady formulation, or condensed uses of chemical materials [7–9]. All over again, it exploits the improved acceptance of nutrients and supplements or enlarged chemicals and bio-chemicals activities [10]. According to [11–13], Nano metals and Nano polymers have been established to “developed stages”, in a sense great engineering outputs. Nanotubes, Nanofibers, and fullerenes continue to increase in output. At moments, progressive, purposeful material is at an “immature” stage with low engineering outputs or at an initial stage, i.e., lately put on the markets or being last verified (for beleaguered medication distribution system) [14].

The subtraction of biological pollutants from waste-water, as well as specifically those ensuing from colors, leftovers the main concern for numerous countries. Dye is a carbon-based compound utilized in numerous industries, like textile, papers, plastic, leather plastic, food, printing as well as pharmaceutical, electroplate, and agricultures [15–17]. It must be renowned that this industry uses a significant quantity of water, and accordingly, their waste-water comprising dye in important quantity is discharged into usual water. Furthermore, these dyes stop penetrating Sun light into waters, reduce photo-synthetic activities and hence cause disturbances of marine equilibriums [18]. It must be stated that deprived of appropriate treatment, this dye can remains in natural waters for an extended time [19]. These are why numerous physical techniques such as adsorptions [20–23], coagulations [24], bio-degradations [25], and several chemical techniques such as chlorination, zonation, etc. [26] have been utilized to decrease the special ecological effects of dye. Nevertheless, physicals, as well as biosynthesis, don’t eliminate contaminants; they only alter them to alternative phases. In the case of chemical techniques, they have the disadvantage of by means of sturdy oxidants like chlorines and ozone, which are themselves contaminants. The further most appropriate ways to eliminate these wastes are their degradation by photo-catalysis. In fact, the dye can be corrupted in the existence of photo-catalysts when exposed to observable lights because of their absorptions of invisible regions.

In previous periods, specific attention has been absorbed to mixed photo-degradations by metallic oxide due to their widespread utility in biological synthesis as well as their ecological application [27, 28]. Amongst the various metallic oxides used, it has been conveyed that Titania (TiO_2_) and zinc oxide (ZnO) is the furthermost stable in chemical reactions and are nontoxic [29, 30]. These are what explain their solicitations in various areas [31, 32]. Titania (TiO_2_) has been extensively investigated as a photo-catalyst and originated from having very respectable photo-catalytic activities, and its applications through solar energies are powerfully restricted by its wider forbidden energy band gap (3.2 eV) and its low quantum efficiencies [33]. So, it is mandatory to use biological methods to produce TiO_2_ nanoparticles.

Additionally, TiO_2_ shows not only antifouling as well as anti-bacterial characteristics but also virtuous photo-catalytic activities [34]. Due to its many welfares, such as great photo-catalytic ability, high oxidizing power, low toxicity and biocompatibility, excellent chemical stability, and ease of accessibility, TiO_2_ is one of the best materials for photo-catalytic processes [35–37]. In this study, the Rosemary (*Rosmarinus officinalis L.)* is an evergreen perennial culinary herb belonging to the family Lamiaceae and is popularly available and used as a spice and medicine. The herb is traditionally used to treat memory-related disorders, hypertension, headache, insomnia, and diseases related to the respiratory system. The essential oil from its leaves is used as a natural antimicrobial, pesticide, and insect repellent [36]. The therapeutic properties of rosemary have been attributed to its phytochemical constituents, such as phenolic acids, flavonoids, and terpenoids. These bioactive compounds act as reducing, capping, and stabilizing agents during nanoparticle formations [37].

In this research, the degradations of dye that are crystalline violets (CV) under Sunlight irradiations by using biosynthesized TiO_2_ nanoparticles from *Rosemary* leaf extracts at 90°C and 200°C were studied.

## Materials and methods

The healthier Rosmarinus leaves were gathered from Dambi Dollo University, Oromia region, Ethiopia. Titanium Iso prop oxide is used as a titanium precursor and sodium-hydroxide acts as pH adjuster, methylenes blues (MB), and crystals violets from BHDs were all compounds utilized and deprived of extra purifications. The plant we used in this report was cultivated in the local area of Dambi Dollo Town, Oromia, Ethiopia. A voucher specimen of the plant (*Rosmarinus officinalis* L.) was confirmed and deposited at Dambi Dollo University, Ethiopia. This study complies with relevant international, national, institutional, and legislative guidelines. The authors took the formal identification of the plant material used in this study.

### Preparations of Leaf extracts

The gathered aqueous and healthier leaves of *Rosmarinus officinalis* L. was splashed in double-distilled waters, tracker dehydrated for 3 to 4 week, and then grounded utilizing blender grinders. For the preparations of fresh leaf extracts (FLE) solution, 20 g of precipitate was liquefied in 150 mL of distilled water and heated at 75^o^C for 20 min to murder the pathogen in FLE solutions. Then after refrigeration, FLE solutions were cleaned through filter papers and kept at 4^o^C for supplementary use.

### Biological synthesization of TiO_2_ nanoparticles through Rosemary (Rosmarinus officinalis) leaf’s extracts

**Fig. 1:**
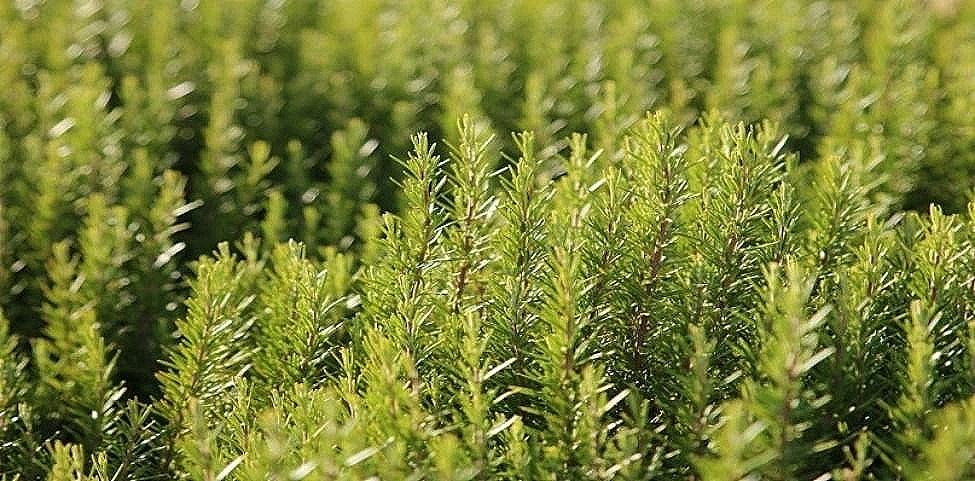
Rosemary (Rosmarinus officinalis) plants were originally taken from Dambi Dollo University, Ethiopia

**Fig. 2:**
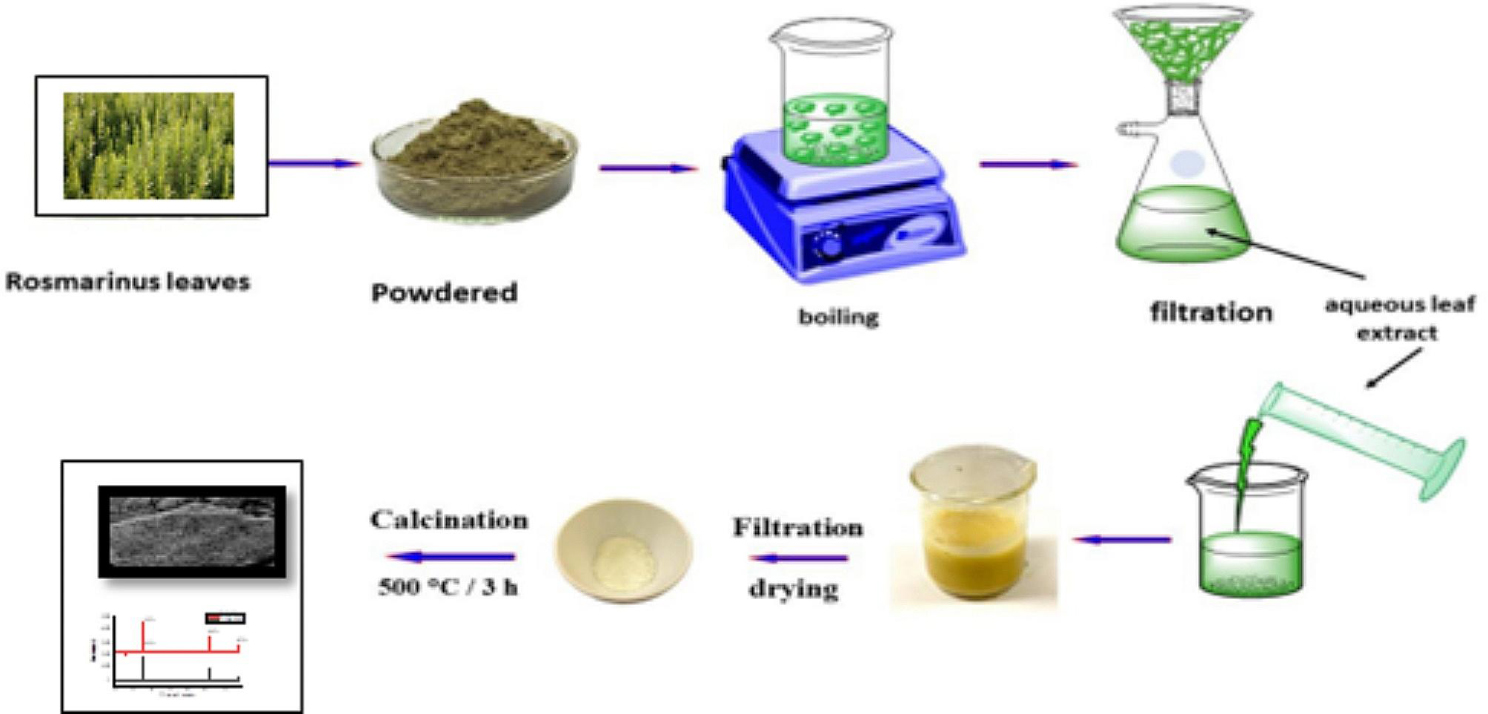
Illustrations of TiO_2_-Nanoprtacles synthesis by Rosmarinus officinalis leaf extracts

Titania (TiO_2_) nanoparticles were prepared by mixing Titanium Iso prop oxide with fresh leaf extracts solutions (FLE). The authors have prepared two samples; the first sample was synthesized by mixing 20 ml of 2 M of sodium hydroxide (NaOH) to a solution established of 10 g Titanium Isopropoxide in 25 ml of the leaf extracts confined in a bottle and Titanium Isopropoxide dissolved in deionized water forming Titanium ion and the solution was colorless. While leaf extracts were added to the solution, a whitish precipitate color formed to confirm the reduction of ions to the nanoparticle. The bioactive active compound available in the leaf extract reduces this ion to form Titanium hydroxide. The bottle comprising the resultant mixtures was closed and heated at 90 °C with a stirring time of 120 min. The catalysts hence synthesized were indicated TiO_2_-90. The second was in similar ways, but in its place of being heated in the bottle, it was transported to a Teflon wrinkled steel autoclave and heated at 200 °C for 120 min. At 200 °C the color of the solution changed automatically and the precipitate formed. The catalysts hence organized were indicated TiO_2_-200. In both circumstances, the gained snowy precipitates were alienated and washed many ways with solutions of double distilled water: ethanols (4:1). Then the samples were kept in an oven at 90 °C and calcinated in a furnace at 450 °C for 4 h, powder form of TiO_2_ Nanoparticles. As shown in Fig. 1 original plant and Fig. 2 illustrate the flow chart of the preparation of TiO_2_ Nanoparticles from Rosemary (*Rosmarinus officinalis*) leaf extracts.

.

### Photo-catalytic tests

A 20 mg of photo-catalyst and 15 ml of 15 ppm of dyes were deferred in bottles, and the mixtures were stirred in the dark to assess adsorption-desorption equilibriums. The photo-degradation was conducted under sunlight on a strong day in April with a temperature of 39 °C. The resultant suspensions were centrifuged at 2500 rpm for 10 min before calculating the absorbances through a UV-Vis spectrophotometer. The photo degradation rates of methylenes blues (MB) were measured by formulae (1) [38]:

1 $${\text{Degradations}}\,\% \, = \,{A_0}\,\left( {{\text{control}}} \right)\, - \,At/{A_0}\,\left( {{\text{control}}} \right)$$

where *A*_0_(control) is a preliminary absorbance of MB; *At* is the absorbances of solutions after Sunlight irradiations at time (t).

### Characterization techniques

**Fig. 3:**
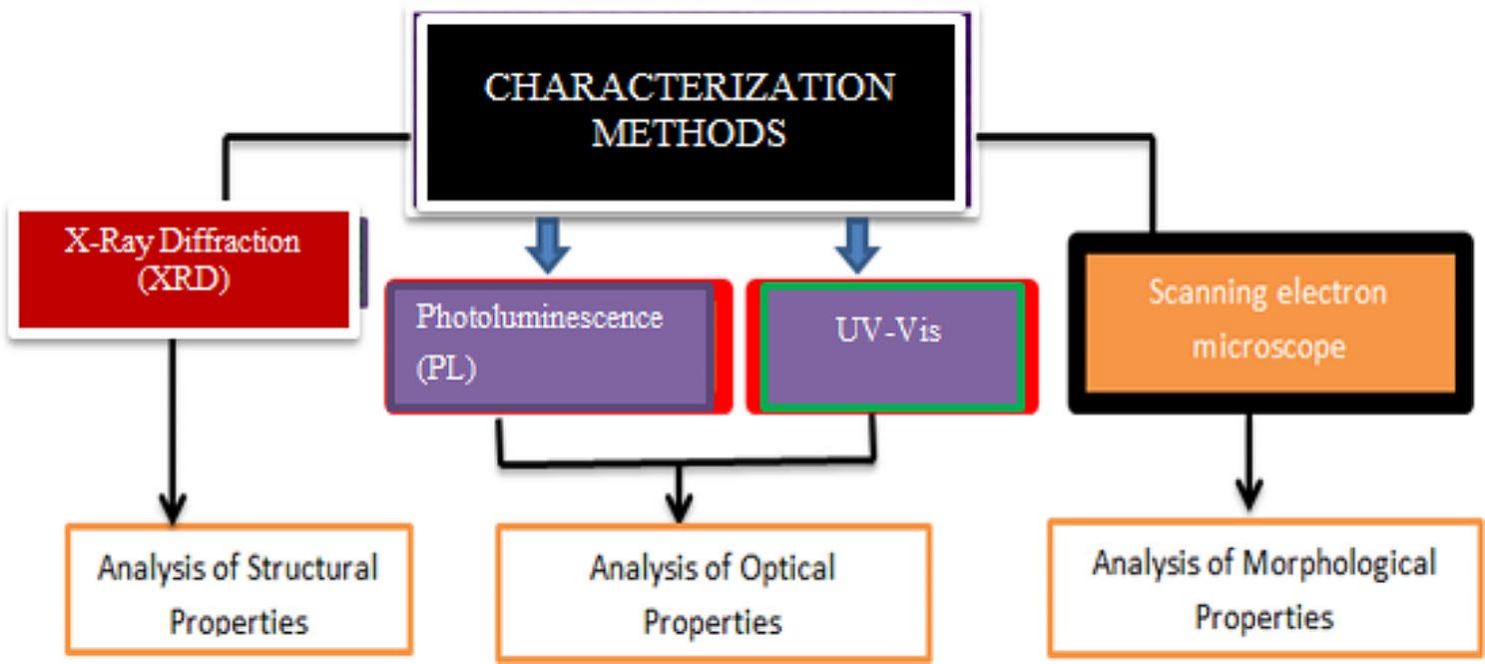
Characterization techniques and their roles

X-Ray Diffraction (XRDs) calibrations were conducted, engaging an Ultimo-IV, X-ray-Rigaku diffractometer through Cu-Kα radiations. UV-Vis and Photoluminescence (PL) spectral characterizations were attained by revenues of double beams UV-Vis spectro-photometer (Philips-8800) and Photoluminescence spectroscopy, respectively. Catalysts Superficial morphologies were studied through scanning electron microscopy (SEM). Figure 3 displays the characterization techniques and their role in the analysis of materials.

## Results and discussion

### Structural characterization of TiO_2_ nanoparticles

**Fig. 4:**
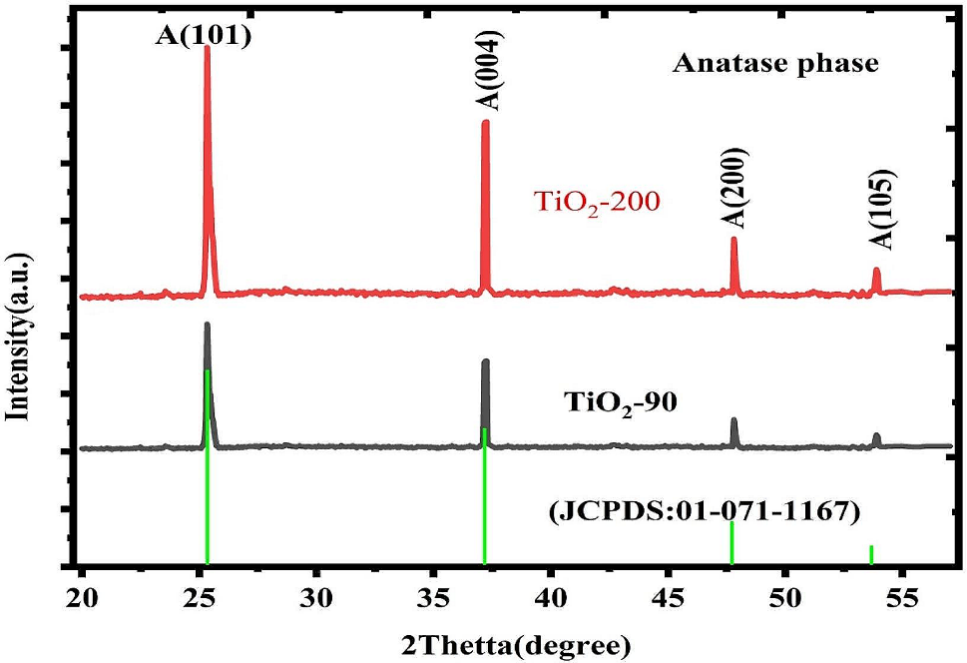
An X-Ray diffractions pattern of TiO2 nanoparticles from Rosmarinus officinalis leaves extracts at temperatures of TiO_2_ at 90° and 200°

In Fig. 4. Displayed XRD configurations gained from the TiO_2_ nanoparticles. The perceived peaks were indexed with a cubic phase configuration as confirmed over a standard (JCPDS N0. 05 01-071-1167), and they are characterized by consistent plane indices in continues. The intense peaks display that constituents have reputable crystalline natures. The synthesized sample is cubic crystals with peak positions 25.37°, 37.19°, 47.81° and 53.89° corresponding (004), (200), and (105) planes respectively. which strengths are mostly liable to preparation time. Additionally, the nonappearance of extra peaks compatible with metallic groups and impurities tells the honorable quality of nanoparticles. Average crystallite sizes calculated for TiO_2_-90 and TiO_2_-200 were 49.11 nm and 41.79 nm respectively by Debye Scherer’s formula [3, 39, 40].

2 $${D}\,=\,\frac{K\lambda }{\beta Cos\theta }$$

Where K is the number around (0.94), $\lambda$ is the wavelength (0.15418 nm), & $\beta$ is the full width at half supreme of a well-definite deflection peak The crystallite sizes entitled nanocrystalline nanoparticles As displayed in Fig. 4, the XRD shows that the synthesized sample is cubic crystals with peak positions 25.37°, 37.19°, 47.81° and 53.89° corresponding (004), (200) and (105) planes respectively. The obtained output shows well-intentioned agreement with previously published works [41–43]. At high temperatures, intense peaks were observed from the XRD graph. Using low temperatures to reduce the rate of the electron-hole recombination processes, trapped electrons, and conduction band electrons exhibit lifetimes. An impression behind times is all nearly protections electrons and holes stable in small areas [44, 45].

TiO_2_ nanoparticles are bio-synthesized, and the nucleation ratios are calculated to be the biggest growth rates due to the plentiful numbers of nucleation centers that depart on the surfaces of the substrate. This can clarify why the TiO_2_ nanoparticles are compressed with a slight size of crystals [46]. Table 1(a) and (b) show the constraints deliberated through Scherer’s equations and XRD data. At high temperatures, higher intense peaks and smaller crystalline sizes were observed from XRD graphs of TiO-200. This shows the preheating of the solutions of nanoparticle formation and structural properties. This reveals the important influences of temperatures on the characteristics of Nanoparticles prepared through biological procedures [47–50].

From XRD data, the crystal parameters were determined and discussed in Table 1.

**Table 1:** (a) Constraints of crystals deliberated using XRDs data for TiO2-90 and (b) TiO2-200

2Thetta(Degree)	FWHM(Radian)	Crystalline size (nm) From XRD	Crystalline size (nm) From TEM
(a)			
25.37099	0.1957	39.60332	30
37.19868	0.13023	57.81598	40
47.81947	0.16939	42.87473	50
53.89554	0.12605	56.18166	60
(b)			
25.37099	0.2957	26.21025	30
37.19868	0.13023	57.81598	40
47.81947	0.26939	26.95924	50
53.89554	0.12605	56.18166	60

### Morphological characterizations

**Fig. 5:**
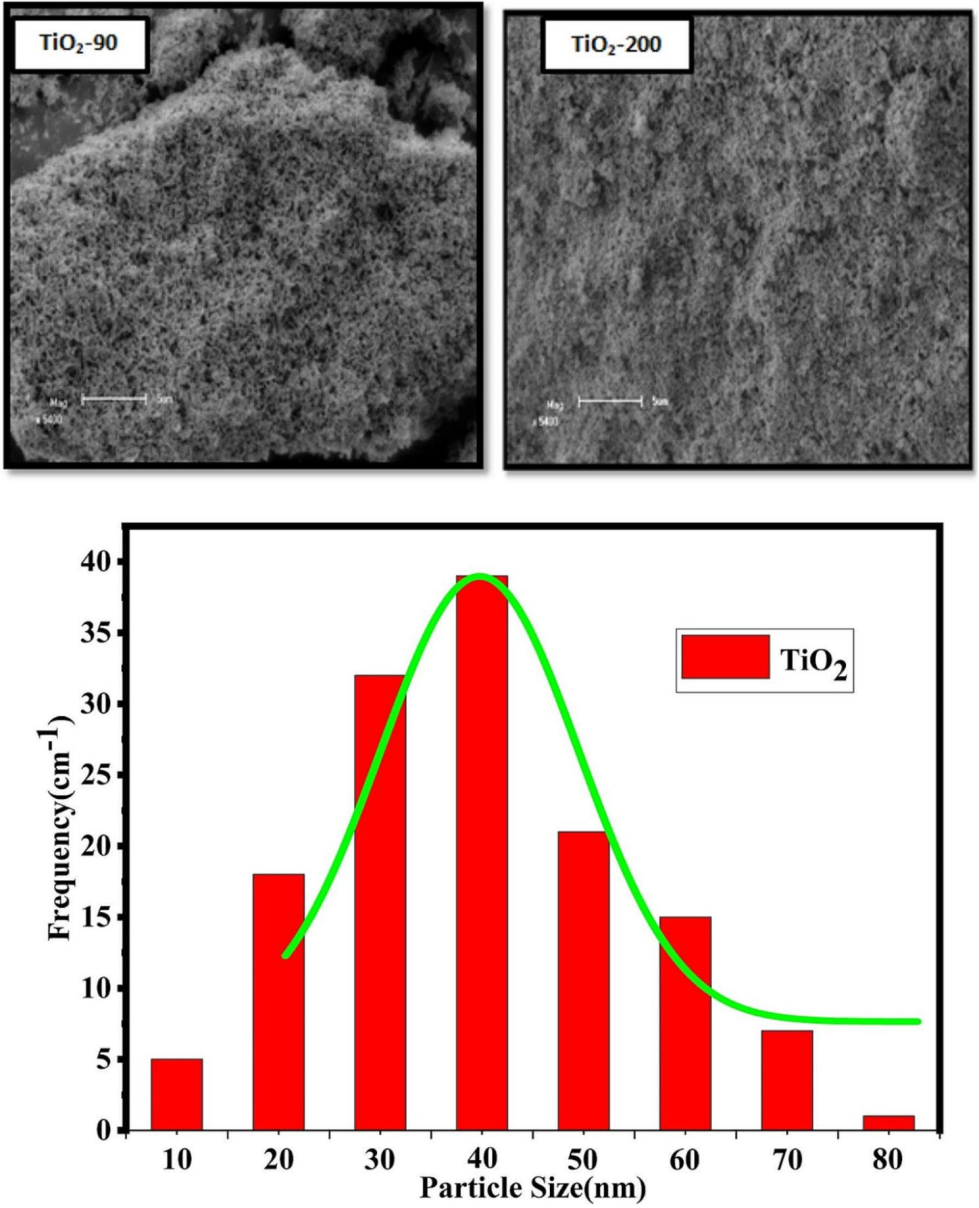
Scanning electron microscope (SEM) micrograph images of TiO_2_ Nanoparticles biosynthesized from Rosemary leaf extracts at 90 and 200°C and particle size distribution

As shown in Fig. 5, Scanning electron microscopy reveals morphologies of TiO_2_-90 and TiO_2_-200 bio-synthesized at 90 °C and 200 °C, respectively. For TiO_2_-90, micrographs show separable crystalline with irregular morphologies and agglomerate cubic-shaped particles. For the other TiO_2_-200 sample, Scanning electron microscopy micro-imaging shows many crumbly agglomerated cubic shapes. As observed for two samples, TiO_2_-90 and TiO_2_-200, the agglomerate particle showed cracks, which may be caused by the discharge of unstable materials during the heating process. At high heating temperatures, more uniform morphology is observed; this shows the influence of temperature in the formation of nanoparticles. This result agreed with the previously reported [51].

**Fig. 6:**
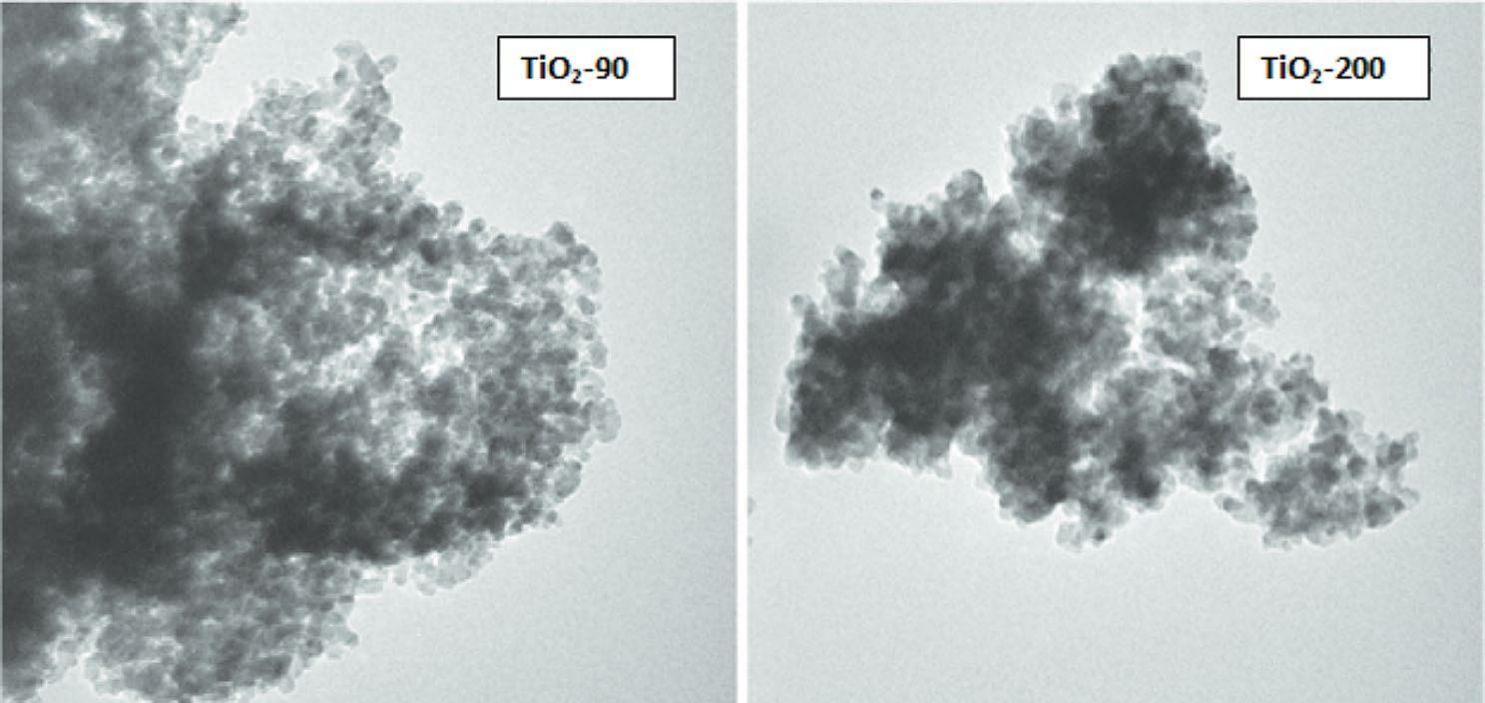
TEM images of green synthesized TiO_2_ nanoparticles at temperatures 90°C and 200°C

**Fig. 7:**
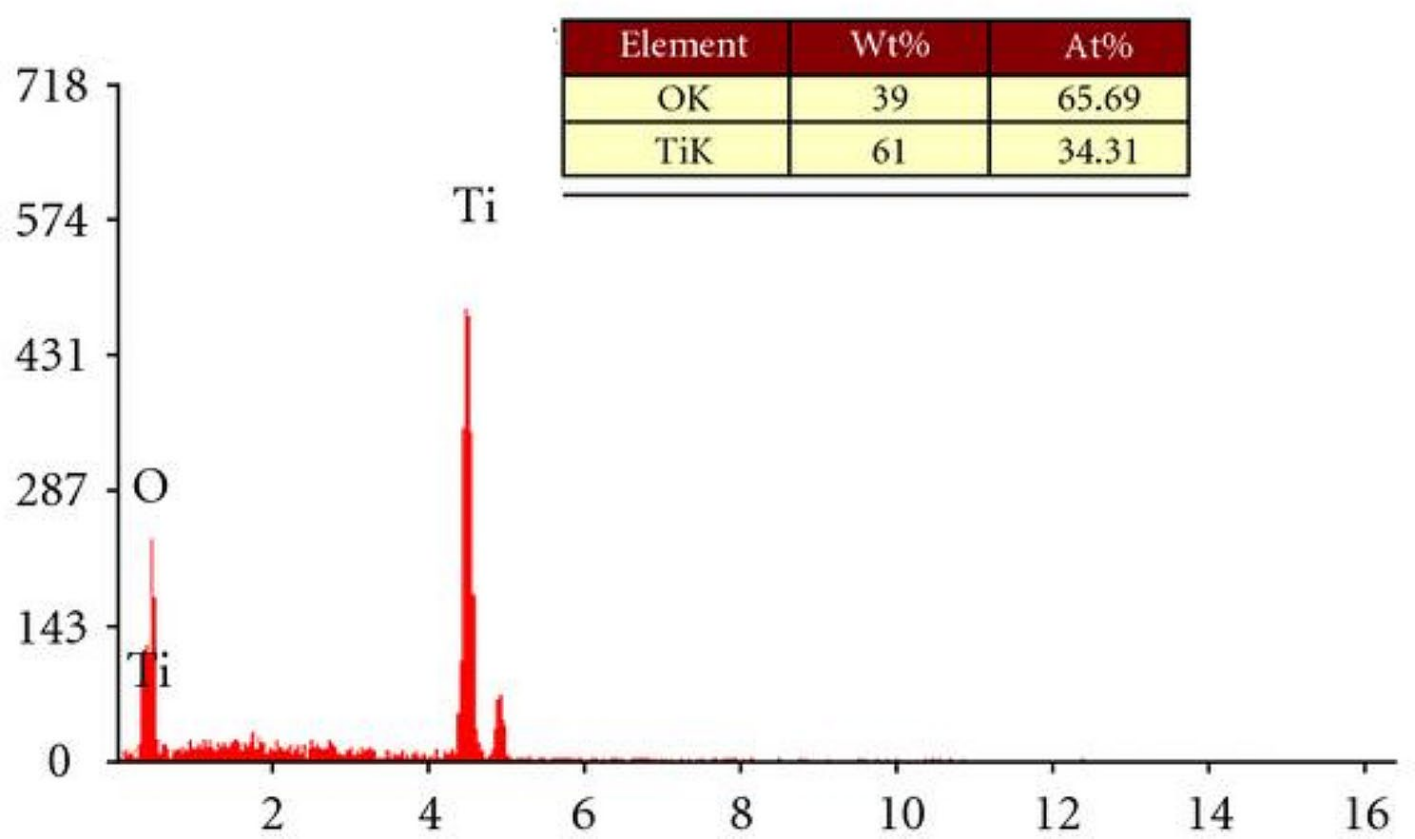
EDS analysis of green synthesized TiO_2_ nanoparticle

TEM analysis was used to determine the morphology, particle size, and particle size distribution of the TiO_2_ nanoparticles, as shown in Figs. 5 and 6. The TiO_2_ samples prepared by the green method at a higher temperature exhibited uniform morphology with adequate particle size distribution. However, at a lower temperature of 90°C, the TiO2 particles exhibited irregular morphology due to the agglomeration of primary particles consisting of either some single particles or clusters of particles. The average size and size distribution of nanoparticles were determined from TEM images using Image tool software, considering at least 100 particles for each sample. The average particle size is about 30 nm and 45 nm at heating temperatures of 90 and 200°C, respectively. These results indicate that the attained particle size increased as temperature increased due to the fact that, as the temperature rose, many adjacent particles tended to fuse together to form larger particle sizes by melting their surfaces. The energy-dispersive x-ray spectroscopy is shown in Fig. 7. Confirming the presence of Ti and O with percentage compositions [52].

### UV-Visible analyzes

UV-visible spectroscopies were conducted to approve the formations of the nanoparticles of TiO_2_ as well as to assess the energy band gap (Eg). The energy band gap of the prepared Nanoparticles was assessed through the Tauc-equation:

3 $$\left( {\alpha hv} \right)1/2{\text{ }} = {\text{ A}}\left( {{\text{hv - Eg}}} \right)$$

**Fig. 8:**
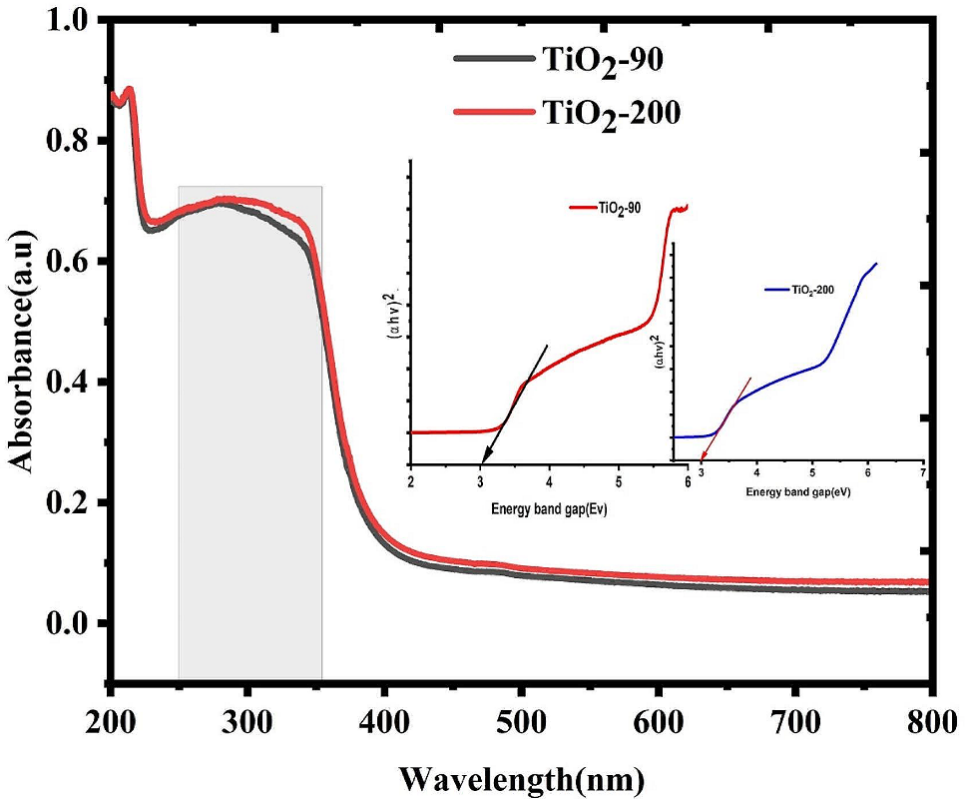
Absorbance and energy gap of TiO_2_-90 and TiO_2_-200 nanoparticles biosynthesized from *Rosmarinus officinalis* leaf extracts at 90 and 200°C

Whereas terms h, ν, α, as well as Eg, denote Planck’s constants, frequencies of wavelength, absorptions coefficients, as well as band-gap energies, respectively. Stands for a proportionate constant, as well as n, which signifies the kind of electron transitions (for direct allowed transition, *n* = 1/2). As could be perceived from the (αhv)^2^ vs. energies plot, Fig. 8 shows the Uv-Vis spectrums of the two samples. TiO_2_-90 and TiO_2_-200 have a band gap of both 3 eV. This energy band-gap is in an array of the stated value of TiO_2_ nanoparticles [51]. The energy band gap of the TiO_2_-90 sample is somewhat more advanced than that of TiO_2_-200. This might be because of the variance in the sizes of their nanoparticles. The variations in energy band-gap can be because of structural parameters and the sizes of grain. In evidence, a sturdy association between absorption peaks and crystalline sizes has been perceived [52]. Thus, this outcome designates that the crystalline sizes of TiO_2_-90 are lesser than those of TiO_2_-200, which is nicely agrees with those of XRD analysis.

### Photoluminescence (PL) spectral analysis

**Fig. 9:**
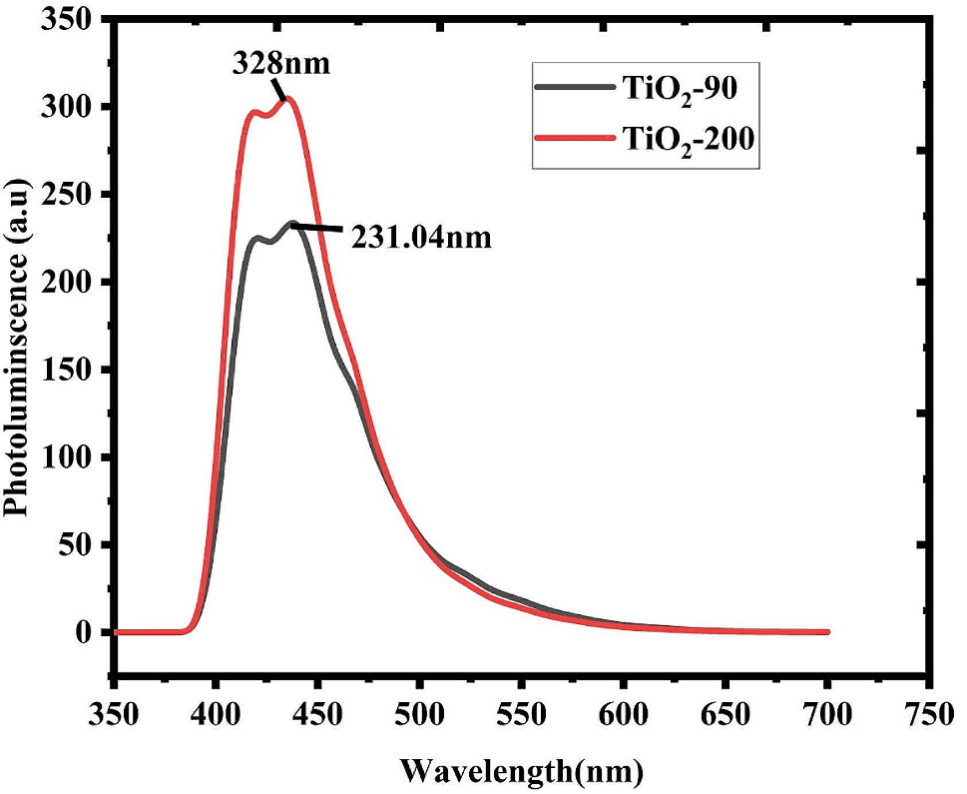
Photoluminescence spectra of biosynthesized TiO_2_ nanoparticles from Rosemary (*Rosmarinus officinalis*) leaf extracts

The optical characteristics of deposited nanoparticles are also investigated by using photo-luminescence. Photo-luminescence spectra of biosynthesized materials were recorded at temperatures 90°C in the ranges of wavelength between 350 nm and 650 nm, as illustrated in Fig. 9. The excitation wavelength lied between 328 and 231.04 nm^56^. The general photoluminescence intensity increases as the temperature vvaries90 to 200°C. The maximum photoluminescence strength at a temperature of 90°C is mostly caused by self-trapped excitons recombination, produced from oxygen vacancies as well as particle sizes which is known as defects center [53, 54]. The photoluminescence intensities decreased progressively with the temperature of 200°C. The behavior of increase and decrement behavior is caused by the lonely phase of anatase. Figure 9 shows new radioactive transitions an occurrence, which leads to a new PL peak at the anatase phase caused by an increment of temperatures [55].

### X-ray photoelectron spectroscopy (XPS)

**Fig. 10:**
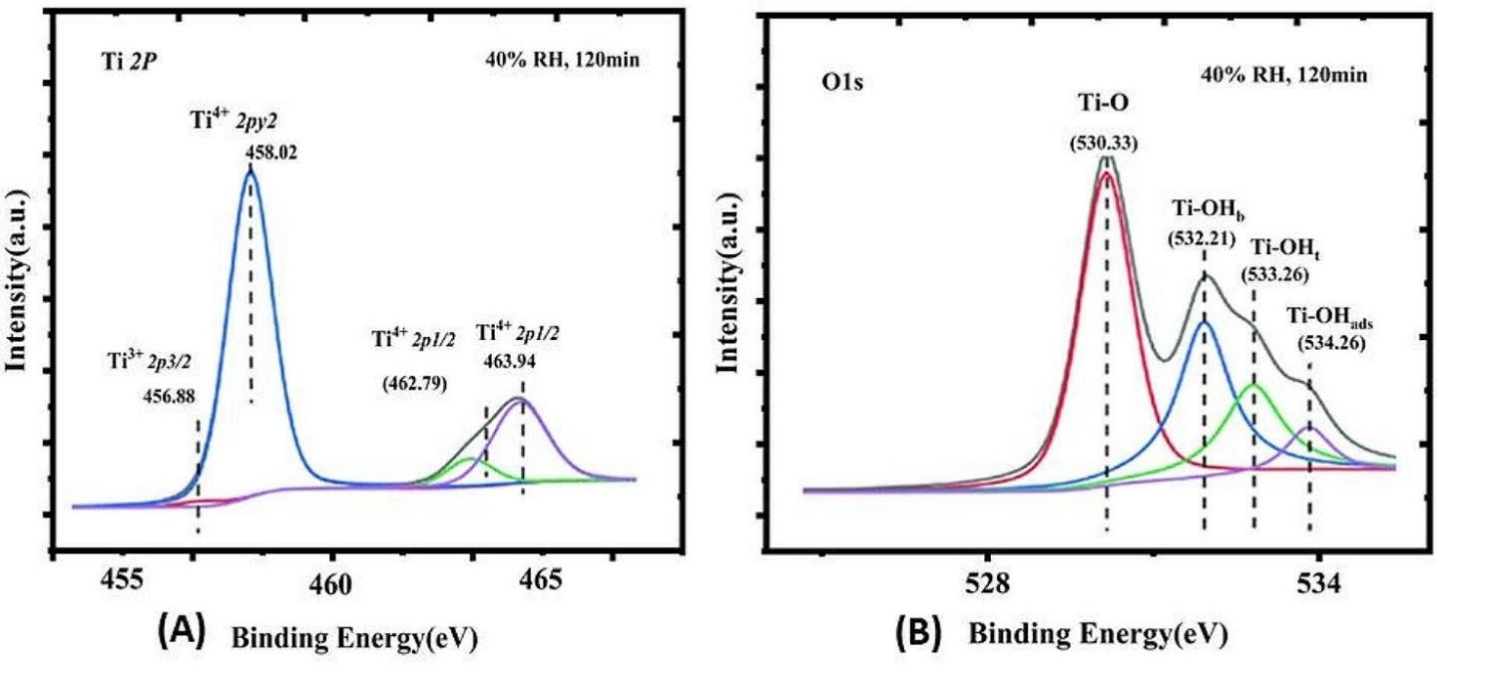
XPS spectra survey of Ti 2p and O 1s peaks of anatase TiO_2_

XPS is a significant technique for the study of the electronic structures of condensed matters and is furthermore an extensively used method for measureable surface analysis. To study the chemical changes that occur during different heating temperature of TiO_2_ nanoparticles, XPS measurements were carried out for TiO_2_-90 and TiO_2_-200 nanoparticles the survey scan spectrums are shown in Fig. 10. The XPS of O and Ti classes on the surfaces of TiO2 nanoparticles (Fig. 10(A) and (B) shows that the O 1s spectrums existed three peaks with binding-energies at 530 eV (lattice oxygen atom, Ti-O), 532.1 eV (terminal-hydroxyl, Ti-OH), and 533.6 eV (surfaces adsorbed H_2_O). Figure 10(B) shows the XPS spectrums of the Ti -2p doublet regions. The peaks of Ti-2p3/2 found at 458.19 eV and Ti-2p1/2 found at 464.12 eV were allotted to Ti^4+^ in TiO_2_. Additionally, the peaks observed at 457 eV of Ti-2p3/2 as well as 462.2 eV of Ti-2p1/2 indicate the existence of Ti^3+^. About 8.7% Ti^3+^ and 90.3% Ti^4+^ were deliberated from the Ti-2p XPS spectrums (Table 2). The absorption of VOs was deliberated as 2.9% based on the assumptions that one-oxygen vacancy is produced with two Ti^3+^. The data are reliable with an earlier report that revealed that the presence of Ti^3+^ in TiO_2_ was convoyed by the formations of VO for preserving the electro-static balances [56].

**Table 2:** Quantitative analysis of oxygen and Ti chemical species

O chemical species	Ti-O	Ti-OH	Water molecule(ads)
	45.99%	34.8	19.21
Ti Chemical species	Ti4+	Ti3+	% of Vo
	90.3%	8.7%	2.9%

### Photo-catalytic activities

**Fig. 11:**
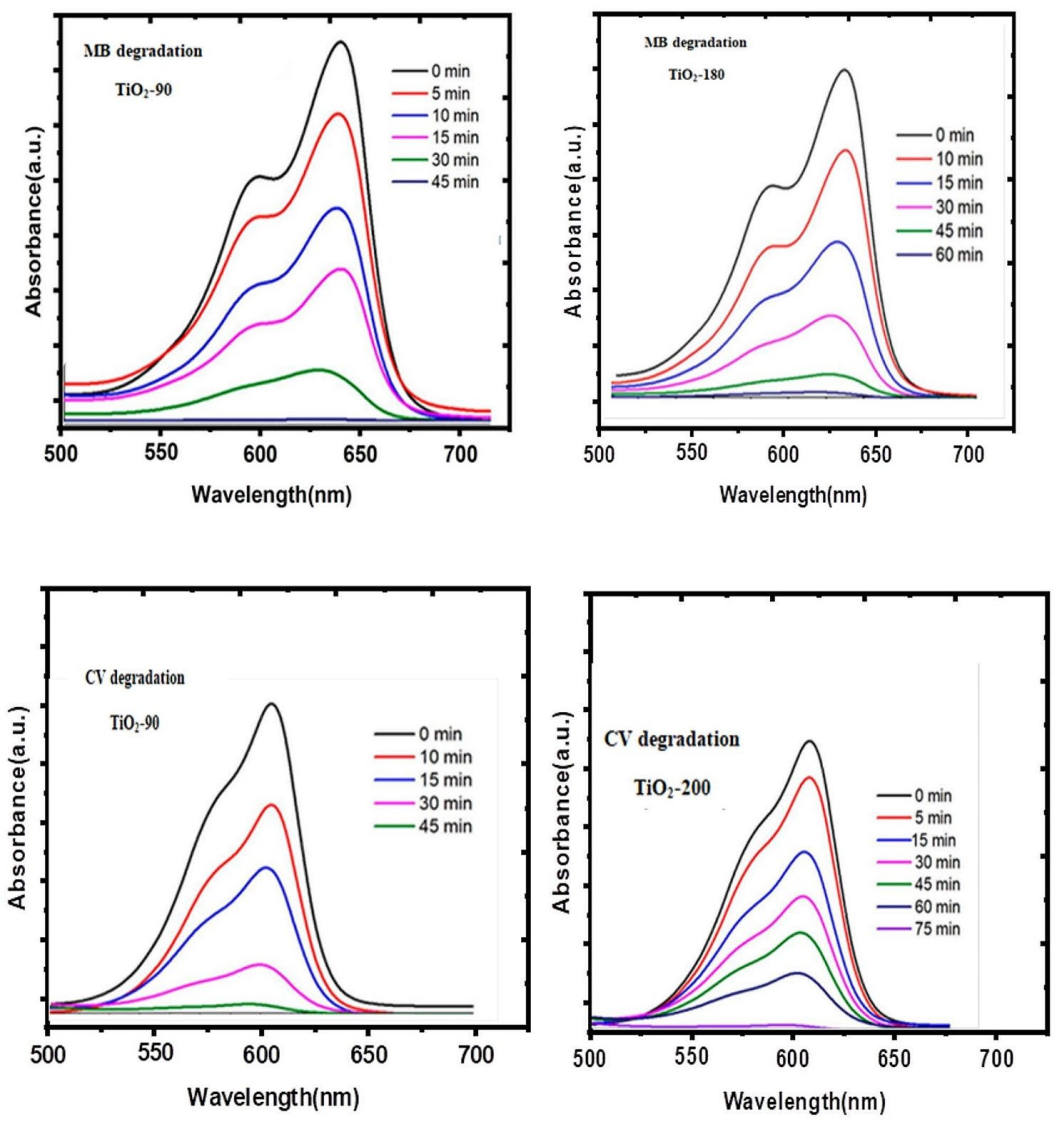
UV–Visible absorptions spectral of degradations of MB with CV by TiO_2_-90 and TiO_2_-200 under Sun light radiations. Reaction condition: Dye concentrations of 20 mg of photo-catalyst and 15 ml of 15 ppm of dye

The photo-catalytic activity of the bio-prepared TiO_2_ nanoparticles was assessed through the photo-degradation of methylene blues (MB) under sun light irradiations. Previous to illuminations, 20 mg photo-catalysts were mixed with the dyes of aqueous solutions (15 mL, 15 ppm). The solutions were stirred in darkness for 25 min to accomplish absorption-desorption equilibriums; then, photo-catalytic reactions began. The photo-catalyst is then visible to the Sunlight for an anticipated time at 45^o^C. Figure 11 displays UV–visible absorption spectra of MB absorbances with reverence to times for TiO_2_-90 and TiO_2_-200.

The solutions of the Methylene blue molecule show two crests, one at 664 similarly at 615 nm, which corresponded correspondingly to monomer and dimer [57]. On irradiations, the peaks at 664 nm have increasingly blue shifts to smaller wavelengths (Fig. 11) due to hypo-chromic effects [58, 59]. In the existence of TiO_2_-90, the absorbances of MB reduced sharply afterward 35 min. Originally the absorption peaks at 664 nm were abundant and superior to the absorption peaks at 615 nm, which provides great alteration between their intensity. After 35 min, these differences are weakened, hence representing that the amount of degradations of monomer is much more sophisticated than that of the dimer [60, 61]. Additionally, the decreases in the intensity of the two crests and a small shift in the direction of the blues of the groups positioned at 664 nm were also perceived. These are instigated by N-de-methylated degradations concomitants with the degradations of phenothiazines [62, 63].

The influences of irradiation time’s discolorations of CVs (Fig. 11) were surveyed properties of a peak at 590 nm, analogous to conjugate tri-phenyl-methane chromospheres. The decreases in absorbances in 590 nm with irradiations are because of the degradations of the chromosphere accountable for the properties of the Colour of CVs. The hypo-chromic shift of peaks at 590 nm of chromosphere at about 575 nm designates N-di-methylation reactions foremost to NO_3_^−^ ions [64, 65]. A comparison of the performance of the TiO_2_ nanoparticles photocatalysts of the current work with another recent work is presented. The outcome clearly reveals that the TiO2 nanoparticles of the current study exhibit dominance in terms of degradation time and efficiencies. In fact, the photo-catalyst degrades almost 100% of MB in a shorter period of time than the TiO2 nanoparticles prepared by using different plant leaf extracts [66, 67].

TiO2 nanoparticles prepared by using different plant leaf extracts [66, 67, 68].

## Conclusion

In this research, the degradations of MB as well as crystal violet (CV) dye by using TiO_2_ Nanoparticles under Sunlight irradiations were studied. Titania (TiO_2_) Nanoparticles were bio-synthesized through *Rosmarinus-officinalis* leaf extracts at 90 °C (TiO_2_-90) and 200$?$(TiO_2_-200). The synthesized materials were characterized using XRD, UV-Vis, PL, SEM, TEM, EDS, and XPS results displayed that bio-synthesis temperatures disturb the shapes and sizes of TiO_2_ nanoparticles. At inferior temperatures, hints to the production of slighter, cubic-shaped, and less-agglomerate crystalline. Photo-catalytic experiments discovered that TiO_2_-90 nanoparticles were well-organized in photo-degradations of MB as well as CV dye likened to TiO_2_-200. The great activities of TiO_2_-90 were because of better Physicochemical characteristics associated with TiO_2_-200. TiO_2_-90 was synthesized by a cheaper and easier process associated with TiO_2_-200, which was synthesized by usual techniques through auto-clave and great temperatures, effectively degrading MB as well as CV dye under photo-light. Photo-degradations of dye under Sunlight as plentifully obtainable energy sources by TiO_2_, synthesized by simpler techniques, can be subjugated to develop an eco-friendly and economical process.

## Data Availability

The datasets generated and/or analyzed during the current study are not publicly available; however, they are available from the corresponding author upon reasonable request.
